# A systematic review of miRNAs as biomarkers for chemotherapy-induced cardiotoxicity in breast cancer patients reveals potentially clinically informative panels as well as key challenges in miRNA research

**DOI:** 10.1186/s40959-022-00142-1

**Published:** 2022-09-07

**Authors:** Cameron Brown, Michael Mantzaris, Elpiniki Nicolaou, Georgia Karanasiou, Elisavet Papageorgiou, Giuseppe Curigliano, Daniela Cardinale, Gerasimos Filippatos, Nikolaos Memos, Katerina K. Naka, Andri Papakostantinou, Paris Vogazianos, Erietta Ioulianou, Christos Shammas, Anastasia Constantinidou, Federica Tozzi, Dimitrios I. Fotiadis, Athos Antoniades

**Affiliations:** 1 Stremble Ventures Ltd, 59 Christaki Kranou, 4042 Limassol, Cyprus; 2 University of Ioannina, 45110 Ioannina, Greece; 3 Bank of Cyprus Oncology Centre, 32 Acropolis Avenue, 2006 Strovolos, Nicosia, Cyprus; 4 European Institute of Oncology, IRCCS and University of Milan, Milan, Italy; 5 National and Kapodistrian University of Athens, Athens University Hospital Attikon, Athens, Greece; 6 2nd Department of Cardiology, Faculty of Medicine, School of Health Sciences, University of Ioannina, and University Hospital of Ioannina, Ioannina, Greece; 7 Department of Oncology-Pathology, Karolinska Institutet and University Hospital, Stockholm, Sweden

**Keywords:** miRNA, microRNA, Cardiotoxicity, Breast cancer, Chemotherapy, Anthracycline

## Abstract

Breast cancer patients are at a particularly high risk of cardiotoxicity from chemotherapy having a detrimental effect on quality-of-life parameters and increasing the risk of mortality. Prognostic biomarkers would allow the management of therapies to mitigate the risks of cardiotoxicity in vulnerable patients and a key potential candidate for such biomarkers are microRNAs (miRNA). miRNAs are post-transcriptional regulators of gene expression which can also be released into the circulatory system and have been associated with the progression of many chronic diseases including many types of cancer. In this review, the evidence for the potential application of miRNAs as biomarkers for chemotherapy-induced cardiotoxicity (CIC) in breast cancer patientsis evaluated and a simple meta-analysis is performed to confirm the replication status of each reported miRNA. Further selection of miRNAs is performed by reviewing the reported associations of each miRNA with other cardiovascular conditions. Based on this research, the most representative panels targeting specific chemotherapy agents and treatment regimens are suggested, that contain several informative miRNAs, including both general markers of cardiac damage as well as those for the specific cancer treatments.

## Introduction

Cardiomyopathies and cardiovascular diseases are well-known side effects of the principle chemotherapy agents used against breast cancer (BC) such as anthracyclines, monoclonal antibodies, alkylating agents and anti-metabolites [1, 2]. As cancer treatment outcomes are improving, progressive cardiac dysfunctions can impact post-treatment survival times [3, 4] to the extent that, following chemotherapy treatment, the risk of death is doubled compared to non-cancer sufferers and this figure is expected to continue increasing with time [4]. There are several established cardiotoxicity risk factors, such as the type of chemotherapy agent, the cumulative dosage and the infusion regime, in addition to patient-related factors such as age, female sex and any pre-existing cardiac or cardiovascular conditions [5]. BC patients are at considerable risk of chemotherapy-induced cardiotoxicity (CIC) through the use of agents such as anthracyclines and HER2-directed monoclonal antibodies although this could be reduced if identified and managed from the outset of treatment [6]. Anthracycline-associated cardiotoxicity can cause injury and death of cardiomyocytes leading to Left Ventricular Dysfunction (LVD) which produces symptoms consistent with heart failure and is often irreversible [7]. Biomarkers of cardiotoxicity include cardiac troponins I and T (cTnI, cTnT), which are released through myocyte necrosis and the N-terminal prohormone of brain natriuretic peptide (NT-proBNP) which is linked to cardiac strain [8]. Elevated levels of these biomarkers have been noted in cardiovascular conditions including cardiotoxicity but require establishing baseline values and repeated measurements [9]. The monitoring of such biomarkers in an oncology setting has not been widely adopted although it is recommended for the management of cancer patients and to initiate cardioprotective treatments where required [10].

Cardiotoxicity is the result of a series of complex reactions to a chemical agent involving mechanisms such as metabolic disorders, oxidative stress, mitochondrial dysfunction, calcium overload, myocardial fibrosis and cardiomyocyte autophagy [11, 12]. Over 200 genes have been identified within the pathway of anthracycline metabolism and transport including several genetic variations affecting cardiotoxicity risk [13]. However, the dynamics of the relationships between chemotherapy treatments and cardiotoxicity pathways are highly complex and the significance of some of these genetic variations are still largely unknown [11]. Changes in gene expression can be regulated by several types of non-coding RNA (ncRNA) consisting of long-non coding RNA (lncRNA), circular RNA (circRNA) and microRNA (miRNA) [14]. microRNAs are short (17–24 nucleotide) RNA sequences which act as modifiers of gene expression by preferentially binding to messenger RNA (mRNA) transcripts either in the 3′-untranslated region (UTR) or coding sequence, leading to inhibition of mRNA translation, protein synthesis and promoting mRNA degradation [15]. Binding of miRNAs to functional mRNA transcripts can be imprecise, involving seed sequences of only 6–8 nucleotides [16] meaning that each miRNA can have multiple mRNA targets, potentially influencing several genes and functional pathways [17]. There are over 2000 miRNAs identified so far in humans and a nomenclature of numbers and letters prefixed with “miR” has been established based primarily on order of discovery and orthologs in other species [18]. In the cell cytoplasm, miRNAs go through a process of maturation with the Dicer RNAse III endonuclease enzyme which lead to subtle differences in their RNA sequences leading to groups of very similar miRNAs, termed families, which may or may not have similar or overlapping functions and mRNA targets [16]. Identical mature miRNAs can also originate from different areas of the genome, for example, miR-7-1 (chromosome 9) and miR-7-2 (chromosome 15). miRNAs can be further classified into isomirs which are changes in sequence length or nucleotides at the 3′ or 5′ ends, sometimes with modifications to function and targets [19].

**Fig. 1:**
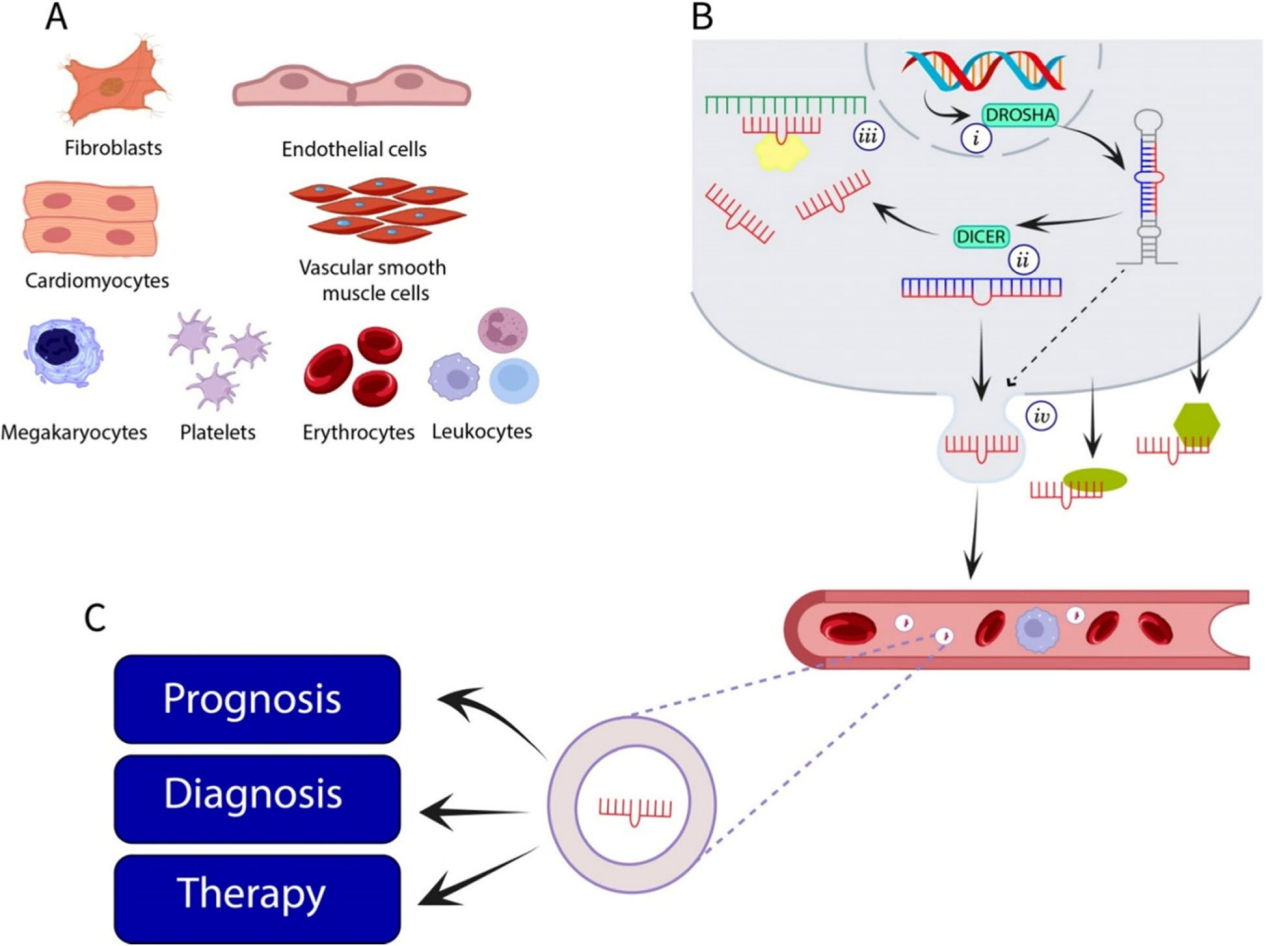
Schematic of circulatory miRNAs. **A** Exosomal microvesicles can be released by several types of cell within the cardiovascular system and enter into the circulatory system. **B** The biogenesis of miRNAs: i) biogenesis begins in the cell nucleus with the transcription of the DNA into large pri-miRNAs which are cleaved by the Drosha enzyme into pre-miRNAs that can be transported into the cytoplasm, ii) The Dicer enzyme cleaves the pre-miRNA into immature miRNA duplexes of 21 to 25 nucleotides and then to a single stranded mature miRNA, iii) miRNAs can be incorporated into a RNA-induced silencing complex (RISC) which can bind or partially-bind to mRNA and inhibit translation or promote degradation, iv) pre-miRNAs and mature miRNAs can be secreted from the cell in exosomes or lipid vesicles as well as bound to RNA-binding proteins and lipoproteins. **C** microvesicles can enter the circulatory system where they can be detected from blood samples (and other bodily fluids) for the purposes of prognosis, diagnosis and as therapeutic targets

Whilst miRNAs are active within the cytoplasm, they can also be secreted into vesicles (exosomes), bound to proteins or lipids and enter the circulatory system where they may potentially facilitate cellular communications [20]. As indicated in Fig. 1, many types of cell within the cardiovascular system including cardiomyocytes, fibroblasts, vascular smooth muscle cells and endothelial cells can release exosomes containing microRNAs [21]. Moreover, several types of blood cells including erythrocytes, platelets, leukocytes and megakaryocytes can also release miRNAs [22, 23] which makes the analysis of blood samples problematic unless prepared carefully [24]. As dysregulation of miRNAs has already been associated with many severe diseases [25], these extracellular miRNAs are of great interest as biomarkers due to their properties of being potentially disease-specific, stable, quantifiable and easily extracted from a range of clinical samples [26]. In this manuscript we review the potential of microRNAs (miRNAs) as biomarkers for chemotherapy-induced cardiotoxicity in BC patients.

## Methods

A systematic literature review was carried out using the PRISMA methodology [27]. The databases of PubMed, Cochrane Central, Embase, Scopus and Google Scholar were systematically searched with the terms ‘chemotherapy induced cardiotoxicity’, ‘breast cancer’, ‘biomarkers’, ‘microRNA’, ‘anthracycline’, ‘trastuzumab’, ‘doxorubicin’ and ‘epirubicin’. The search was restricted to articles in English between the years of 2000–2022 and only considered miRNAs linked to cardiotoxicity in clinical studies of breast cancer patients. Review papers, letters and editorial articles as well as studies in animal models and stem cells were excluded. This search resulted in 922 papers of which 166 were considered relevant and were studied at the abstract level by two authors (CB, AA). From these articles, eight were examined in full and selected for inclusion in this manuscript.

In order to further clarify the function of each of the miRNAs shortlisted by the review in other cardiovascular conditions, the literature was further searched for human clinical studies on each individual miRNA qualified by the terms ‘mir’, [‘miR number’], ‘microRNA’, ‘cardio*’, ‘coronary’, ‘heart’, ‘human’. This review of miRNA functions was limited to only clinical studies in cardiovascular diseases, where a significant change in expression of the relevant miRNA was reported. A total of 656 potentially relevant publications were screened and from these, 143 were selected based on their relevance. These titles were studied at the abstract level by two authors (CB, AA) and the papers selected were examined in full by both authors. Review papers, letters and editorial articles as well as studies in animal models and stem cells were excluded. A total of 104 articles were studied in full and from these, 90 were selected for inclusion.

**Fig. 2:**
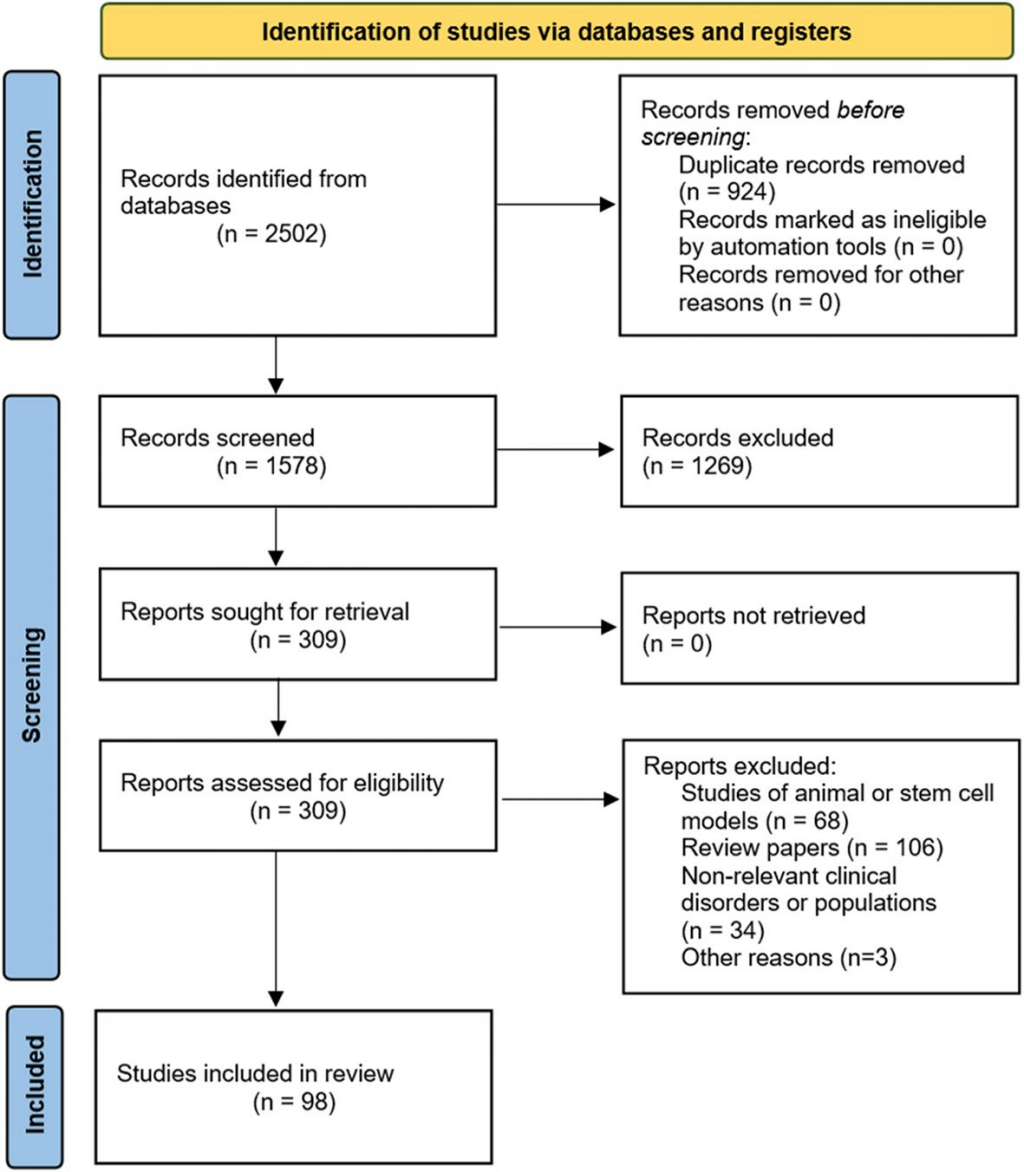
PRISMA schema [26] for the systematic review of literature

Figure 2 shows the PRISMA diagram for the literature review as a whole with 1578 papers being identified, from which 309 were shortlisted and 112 reviewed in full. Based on the criteria outlined above, 98 papers were selected for inclusion in this manuscript.

## Results

Clinical studies of breast cancer patients indicate that dysregulation of several miRNAs have been noted following anthracycline treatment [28–35], as summarised in Table 1. Many of the miRNAs examined were related to functions associated with cardiac damage such as apoptosis, hypertrophy and inflammatory responses which suggests that they could be used as potential biomarkers for the incidence of anthracycline induced cardiotoxicity. Table 1 is divided into four sections based on the strength of evidence for changes in expression in breast cancer patients after chemotherapy. Section A includes miRNAs which have been reported statistically significant in only one study, Section B includes miRNAs which have been reported statistically significant and replicated in other studies whether significant or not. Section C includes miRNAs which have been reported as statistically non-significant and in Section D those miRNAs are reported which although were found to have statistically significant findings, they also showed contradictory expression change directions in independent studies.

**Table 1:** List of microRNA’s targeted by studies of breast cancer patients displaying cardiotoxicity following treatment with Anthracyclines and comparison of expression with control groups

MicroRNA	Reference	Subjects (Healthy Controls/ Chemotherapy group)	Treatment	Period Tested	Expression Change to Control group	Differential Expression	Proposed Role
Section A							
hsa-miR-29a-3p	[33]	17/17	DOX	6 months	Increased	+ 5 RE	Cardiac repair
hsa-miR-199a-3p	[28]	38/7	NAC	2 cycles	Increased	+ 1.2 FC	Cardiomyocyte regeneration
hsa-miR-1273 g-3p	[34]	20/20	AC	PT	Decreased	−0.52Log_2_ΔCt	Regulatory function of TGF-β pathway
hsa-miR-4638-3p	[34]	20/20	AC	PT	Decreased	−1.37 Log_2_ΔCt	Regulatory function of TGF-β pathway
Section B							
hsa-miR-34a-5p	[33]	17/17	DOX	6 months	Increased	+ 40 RE	Cardiac repair
	[28]	38/7	NAC	2 cycles	Increased	+ 24.3 FC	
	[32]	44/12	DOX	3 months	No significant change	–	
	[32]	14/18	EPI	3 months	No significant change	–	
hsa-miR-1	[31]	46/10	DOX	4 cycles	Increased	+ 2Log_2_FC	Cardiac hypertrophy
	[28]	38/7	NAC	3 months	No significant change	–	
	[32]	44/12	DOX	3 months	No significant change	–	
	[32]	14/18	EPI	3 months	No significant change	–	
hsa-miR-17-5p	[29]	170/9	EC-D	8 cycles	No significant change	–	Pro-angiogenic
	[30]	346/19	EC-D	8 cycles	Decreased	0.213 OR	
hsa-miR-19a	[29]	170/9	EC-D	8 cycles	Increased	+ 2.1 RE	Pro-angiogenic
	[30]	346/19	EC-D	8 cycles	No significant change	–	
hsa-miR-122-5p	[32]	44/12	DOX	3 months	Increased	+ 3 ΔΔCt	Coronary disease
	[32]	14/18	EPI	3 months	No significant change	–	
hsa-miR-130a	[29]	170/9	EC-D	8 cycles	No significant change	–	Cardiomyopathy
	[30]	346/19	EC-D	8 cycles	No significant change	–	
	[35]	60/12	EC-D + T	15 months	Increased	+ 4 RE	
hsa-miR-378	[29]	170/9	EC-D	8 cycles	No significant change	–	Pro-angiogenic
	[30]	346/19	EC-D	8 cycles	Decreased	0.278 OR	
hsa-miR-423	[28]	38/7	NAC	3 months	Increased	+ 1.3 FC	Progressive heart failure
	[31]	46/10	DOX	4 cycles	No significant change	–	
	[33]	17/17	DOX	6 months	Increased	+ 6.5 RE	
hsa-miR-499	[32]	44/12	DOX	3 months	Increased	+ 2 ΔΔCt	Acute myocardial infarction
	[28]	38/7	NAC	3 months	No significant change	–	
	[32]	14/18	EPI	3 months	No significant change	–	
	[33]	17/17	DOX	6 months	Increased	+ 15 RE	
hsa-miR-885-5p	[32]	44/12	DOX	3 months	Increased	+ 2 ΔΔCt	Liver toxicity
	[32]	14/18	EPI	3 months	No significant change	–	
Section C							
hsa-Let-7b	[29]	170/9	EC-D	8 cycles	No significant change	–	Pro-angiogenic
	[30]	346/19	EC-D	8 cycles	No significant change	–	
hsa-miR-17-3p	[29]	170/9	EC-D	8 cycles	No significant change	–	Cardiac hypertrophy
	[30]	346/19	EC-D	8 cycles	No significant change	–	
hsa-miR-18a	[29]	170/9	EC-D	8 cycles	No significant change	–	Oncogenic inhibitor
	[30]	346/19	EC-D	8 cycles	No significant change	–	in breast cancer
hsa-miR-19b-1	[29]	170/9	EC-D	8 cycles	No significant change	–	Inflammatory
	[30]	346/19	EC-D	8 cycles	No significant change	–	response
hsa-miR-92a	[29]	170/9	EC-D	8 cycles	No significant change	–	Pro-angiogenic
	[30]	346/19	EC-D	8 cycles	No significant change	–	
hsa-miR-133a	[28]	38/7	NAC	3 months	No significant change	–	Acute myocardial infarction
hsa-miR-133b	[31]	46/10	DOX	4 cycles	No significant change	–	Acute myocardial
	[28]	38/7	NAC	3 months	No significant change	–	infarction
hsa-miR-146a	[31]	46/10	DOX	4 cycles	No significant change	–	Inflammatory response
hsa-miR-208a	[28]	38/7	NAC	3 months	No significant change	–	Cardiomyocyte
	[31]	46/10	DOX	4 cycles	No significant change	–	damage
hsa-miR-208b	[28]	38/7	NAC	3 months	No significant change	–	Cardiomyocyte
	[31]	46/10	DOX	4 cycles	No significant change	–	damage
hsa-miR-296	[29]	170/9	EC-D	8 cycles	No significant change	–	Angiogenesis
	[30]	346/19	EC-D	8 cycles	No significant change	–	
Section D							
hsa-miR-20a	[29]	170/9	EC-D	8 cycles	Increased	+ 1.1 RE	Pro-angiogenic
	[30]	346/19	EC-D	8 cycles	Decreased	0.264 OR	
hsa-Let-7f	[29]	170/9	EC-D	8 cycles	Increased	+ 1.1 RE	Pro-angiogenic
	[30]	346/19	EC-D	8 cycles	Decreased	0.228 OR	
hsa-miR-126	[29]	170/9	EC-D	8 cycles	Increased	+ 1.5 RE	Pro-angiogenic
	[33]	17/17	DOX	6 months	Increased	+ 28 RE	
	[30]	346/19	EC-D	8 cycles	Decreased	0.358 OR	
	[28]	38/7	NAC	3 months	Increased	+ 1.3 FC	
hsa-miR-210	[29]	170/9	EC-D	8 cycles	Increased	+ 1.2 RE	Pro-angiogenic
	[30]	346/19	EC-D	8 cycles	Decreased	0.475 OR	

Part A: microRNAs with significant changes in expression with no independent replication, Part B: microRNAs with significant changes in expression and independent replication (whether significant or not), Part C: microRNAs with no significant changes in expression detected, Part D: microRNAs with contradictory evidence of direction of expression change in independent replication. Key: EC-D = Epirubicin + Cyclophosphamide (4 cycles) followed by Docetaxel (4 cycles), DOX = Doxorubicin, EPI = Epirubicin, NAC = Cyclophosphamide + Epirubicin (4 cycles) followed by Paclitaxel (9 to 12 weeks), AC = Anthracycline chemotherapy (not specified), PT = Post-treatment, RE = Relative Expression, FC = Fold Change, OR = Odds Ratio

The evidence from Table 1 indicates that of the 33 miRNAs investigated in these clinical studies, only 14 can be considered as potentially informative for study of cardiotoxicity as they have some indication of a significant change in expression and no contradictory results in replicated studies (Table 1, Sections A and B). The remaining 19 miRNAs have either no evidence of significant expression changes in cardiotoxicity or contradictory evidence of the direction of the expression change and were not considered further.

Within all the clinical studies reviewed, the number of patients diagnosed with cardiotoxicity was low, reaching a maximum of 20 patients. As shown in Table 2, the identification of cardiotoxicity was predominantly based on reductions in left ventricular ejection fraction (LVEF) during or after chemotherapy based on echocardiograms, which conforms to the European Society of Cardiology diagnostic methodology for identifying cardiotoxicity [5]. Other indicators of cardiotoxicity such as heart failure and acute coronary syndrome were rare, whereas, fatal arrythmias were more common but identified in only one study. The use of cardiac Troponin levels to establish cardiotoxicity was used by one study (Table 2). Exclusion criteria for patients in all the studies included a prior history of cardiovascular events such as coronary heart disease, myocardial infarction and heart failure as well as metastatic breast cancer, severe hepatic or renal dysfunction and pregnancy. Three studies included prior chemotherapy or radiotherapy as an exclusion criterion, however, no study provided information for exposure to radiation therapies prior to or during the study period.

**Table 2:** Characterisation of cardiotoxicity in breast cancer patients during or after chemotherapy for each of the reviewed studies

	Reference	[28]	[29]	[30]	[31]	[32]	[33]	[34]	[35]
	Cohort size	45	179	363	56	56/32	34	40	72
	Treatment regime	NAC	ECD	ECD	DOX	EPI/DOX	DOX	AC	ECD-T
Assessment of Cardiotoxicity	Heart failure	1^a^	1	0	0	0/0	0	0	0
	Acute coronary syndrome	0	0	0	0	0/0	0	0	1
	Fatal arrhythmias	0	9	0	0	0/0	0	0	0
	Decline of LVEF ≥10% of baseline or below 53%	17	9	19	10	0/0	4	20	12
	Elevated Troponin level	0	0	0	0	12/18	0	0	0

Key: EC-D = Epirubicin + Cyclophosphamide (4 cycles) followed by Docetaxel (4 cycles), DOX = Doxorubicin, EPI = Epirubicin, NAC = Cyclophosphamide + Epirubicin (4 cycles) followed by Paclitaxel (9 to 12 weeks), AC = Anthracycline chemotherapy (not specified), LVEF = Left ventricular ejection fraction

^a^one patient was diagnosed with heart failure after the study period

**Fig. 3:**
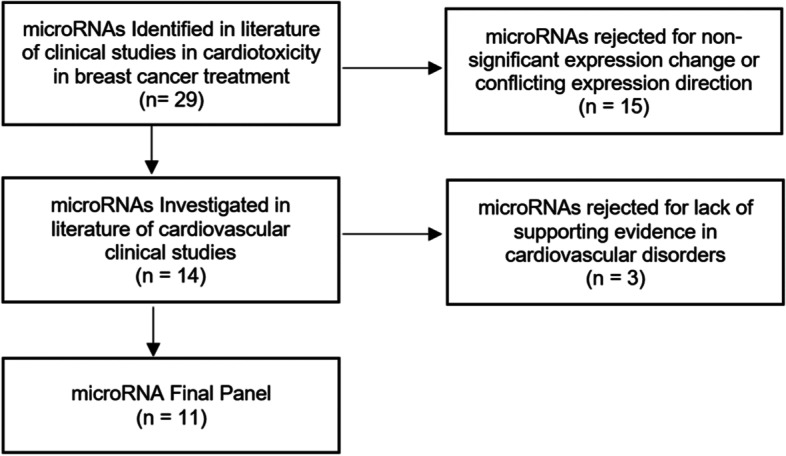
Review of microRNAs associated with cardiotoxicity in breast cancer treatment

The pool of informative markers (Table 1, section A and B) was investigated individually in the literature to identify significant changes in expression linked to other cardiovascular diseases and are discussed in detail below and summarised and in Table 3. A total of 90 relevant studies were identified reporting significant expression changes in these specific miRNAs. Several miRNAs were associated with a wide variety of cardiovascular conditions and have been studied extensively often with a broad agreement in expression direction. Where differences occur, they may be related to the disease or to the methodology employed, for example, some studies report miRNA expression from tissue samples rather than blood plasma which are known to often diverge. Three miRNAs (miR-885, 1273 and 4638) were not associated with any cardiovascular conditions in the literature to date and, therefore, they were not considered suitable for the final panel of informative miRNAs (Fig. 3).

**Table 3:** Supporting evidence for the informative microRNA’s from clinical studies of cardiovascular disorders, indicating the sample type, methodology, cardiovascular condition and the direction of miRNA expression

miRNA	Reference	Sample Type	Evaluation Method	Cardiovascular condition	Expression Change to controls
miR-1-3p	[36]	Plasma	qPCR	Acute myocardial infarction	Increased
	[37]	Serum + urine	qPCR	Acute myocardial infarction	Increased
	[38]	Plasma	NGS	Acute myocardial infarction	Increased
	[39]	Plasma	qPCR	Acute myocardial infarction	Increased
	[40]	Plasma	qPCR	Acute myocardial infarction	Increased
	[41]	Plasma	qPCR	Acute myocardial infarction	Increased
	[42]	FFPE myocardial tissue	qPCR	Sudden cardiac death	Increased
	[43]	Plasma	NGS + qPCR	Failed myocardial reperfusion	Increased
	[44]	PBMC	qPCR	Acute viral myocarditis	Increased
	[45]	Plasma	qPCR	Hypertrophic cardiomyopathy	Increased
	[46]	Cardiac tissue	qPCR	Hypertrophic cardiomyopathy	Decreased
	[47]	Endomyocardial biopsies	qPCR + microarray	Dilated cardiomyopathies	Increased
	[48]	Serum	qPCR	Transcoronary ablation of septal hypertrophy (TASH)	Increased
	[49]	Myocardial tissue	NGS	Tetralogy of Fallot	Decreased
	[50]	Plasma	qPCR	Takotsubo cardiomyopathy	Increased
	[51]	PBMC	qPCR	Hypertensive heart disease	Increased
	[52]	Plasma	qPCR	Non-ST elevation myocardial infarction (NSTEMI)	Increased
	[53]	Plasma	qPCR	Acute coronary syndrome	Increased
	[54]	Right arterial appendage biopsies + Plasma	qPCR	Atrial fibrillation	Increased
	[55]	Myocardial tissue	qPCR + miRNA array	Atrial fibrillation	Increased
	[56]	Myocardial tissue	qPCR	Heart failure	Decreased
	[57]	Serum	qPCR	Heart failure	Decreased
	[58]	Plasma	qPCR	Acute heart failure	Decreased
miR-17-5p	[59]	Plasma	qPCR	Acute coronary syndrome	Increased
	[60]	Plasma	dPCR	Coronary artery disease	Increased
	[61]	Plasma	qPCR	Heart failure	Decreased
	[62]	Plasma	qPCR	Hypertrophic cardiomyopathy	Increased
	[63]	Whole blood	qPCR	Bicuspid aortic valve disorder	Decreased
miR-19a	[64]	Lung tissue	microarray	Pulmonary arterial hypertension	Increased
	[65]	Serum	qPCR	Acute coronary syndrome	Increased
	[66]	Serum	microarray	Atherosclerosis	Increased
miR-29a-3p	[67]	Ascending aorta tissue	qPCR	Bicuspid aortic valve disorders	Decreased
	[68]	Serum	qPCR	Hypertrophic cardiomyopathy	Increased
	[69]	Serum	qPCR	Hypertrophic cardiomyopathy	Increased
	[62]	Plasma	qPCR	Hypertrophic cardiomyopathy	Increased
	[70]	Plasma	NGS + FirePlex assay	Coronary heart disease	Increased
	[71]	Plasma	qPCR	Coronary heart disease	Increased
	[72]	Cardiac valve tissue	qPCR	Valvular heart disease	Decreased
	[73]	Plasma	qPCR	Cardiac fibrosis	Increased
	[74]	Plasma	qPCR	Left ventricular remodelling	Increased
	[75]	Plasma	qPCR	Pulmonary arterial hypertension	Increased
miR-34a-5p	[76]	Plasma	qPCR	Chronic heart disease	Increased
	[77]	Whole blood	qPCR	Cardiac aging	Increased
	[78]	Plasma	qPCR	Left ventricular (LV) remodelling	Increased
	[79]	Plasma	qPCR	Left ventricular dysfunction	Increased
	[80]	Plasma	qPCR	Heart failure	Increased
	[81]	Serum	qPCR	Acute myocardial infarction	Increased
	[82]	Serum	qPCR	Arterial fibrillation	Increased
miR-122-5p	[83]	Serum	qPCR	Coronary artery disease	Increased
	[84]	Plasma	microarray + qPCR	Acute coronary syndrome	Increased
	[85]	Plasma	qPCR	Cardiogenic shock	Increased
	[86]	Whole blood	qPCR	Cardiogenic shock	Increased
	[87]	Plasma	qPCR	Ventricular fibrillation sudden cardiac arrest	Increased
	[88]	Plasma	qPCR	Chronic systolic heart failure	Increased
	[89]	Plasma	microarray	Aortic valve dysfunction	Decreased
	[90]	Ascending aorta tissue + plasma	qPCR	Bicuspid aortic valve disease	Decreased
	[91]	Myocardial tissue	NGS + qPCR	Arrythmogenic cardiomyopathy	Increased
	[92]	Serum	qPCR	Acute myocardial infarction	Increased
	[93]	Serum	microarray	Congestive heart failure	Increased
miR-130a	[59]	Plasma	qPCR	Acute coronary syndrome	Increased
	[94]	Plasma	qPCR	Peripartum cardiomyopathy	Increased
	[95]	Whole blood	microarray	Pulmonary hypertension	Increased
	[96]	Plasma	qPCR	Coronary heart disease	Decreased
	[89]	Plasma	microarray	Aortic valve dysfunction	Increased
miR-199a-3p	[97]	Plasma	qPCR	Acute heart failure	Decreased
	[98]	Right arterial appendage biopsies	qPCR	Postoperative atrial fibrillation	Decreased
	[99]	Plasma	qPCR	Coronary heart disease	Decreased
	[100]	Right arterial appendage biopsies	qPCR	Coronary heart disease	Decreased
	[101]	Plasma	qPCR	Stable coronary artery disease	Decreased
	[102]	Plasma	qPCR	Peripheral arterial disease	Decreased
	[103]	Plasma	microarray + qPCR	Atrial fibrillation in HFrEF patients	Increased
	[104]	Plasma	NGS	Acute myocardial infarction	Increased
miR-378	[105]	Plasma	qPCR	Coronary heart disease	Decreased
	[106]	Whole blood	microarray + qPCR	Coronary artery disease	Decreased
miR-423	[107]	Plasma	qPCR	Acute myocardial infarction	Increased
	[108]	Plasma	qPCR	Dilated cardiomyopathy	Increased
	[109]	Plasma	microarray	Heart failure	Increased
	[58]	Plasma	qPCR	Acute heart failure	Decreased
	[110]	Plasma	qPCR	Acute heart failure	Increased
	[111]	Serum	qPCR	Coronary artery disease	Decreased
	[38]	Plasma	NGS	Acute myocardial infarction	Increased
	[112]	Plasma	qPCR	Acute myocardial infarction	Increased
	[113]	Plasma	qPCR	Cardiogenic shock	Increased
	[114]	Plasma	qPCR	Left ventricular remodelling	Increased
	[115]	Serum	qPCR	Left ventricular remodelling	Increased
	[116]	Whole blood	microarray + qPCR	Transposition of the great arteries	Increased
	[117]	Serum	qPCR	Heart failure	Increased
	[80]	Plasma	qPCR	Heart failure	Increased
miR-499	[38]	Plasma	NGS	Acute myocardial infarction	Increased
	[41]	Plasma	qPCR	Acute myocardial infarction	Increased
	[118]	Plasma	qPCR	Acute myocardial infarction	Increased
	[119]	Whole blood	qPCR	Acute myocardial infarction	Increased
	[120]	Serum	dPCR+qPCR	Stable coronary artery disease	Increased
	[40]	Plasma	qPCR	Acute coronary syndrome	Increased
	[121]	Plasma	qPCR	Acute coronary syndrome	Increased
	[115]	Serum	qPCR	ST-segment-elevation myocardial infarction (STEMI)	Increased
	[52]	Plasma	qPCR	Non-ST elevation myocardial infarction (NSTEMI)	Increased
	[122]	Endomyocardial biopsies	qPCR	Dilated cardiomyopathy	Increased
	[123]	PBMC	qPCR	Heart failure with preserved ejection fraction (HFpEF)	Increased
	[42]	FFPE myocardial tissue	qPCR	Sudden cardiac death	Increased
	[83]	Serum	qPCR	Unstable coronary artery disease	Increased
miR-885-5p				No relevant associations	
miR-1273 g-3p				No relevant associations	–
miR-4638-3p				No relevant associations	–

*PBMC* Peripheral blood mononuclear cells, *FFPE* Formalin-fixed paraffin-embedded tissue, *NGS* Next generation sequencing, *qPCR* Quantitative polymerase chain reaction, *dPCR* Digital polymerase chain reaction

### Hsa-miR-1

miR-1 is highly expressed in cardiac myocytes and is associated with regulating angiogenesis, cell apoptosis and endothelial functioning. It has a regulatory role on many genes such as the heat shock protein 60 (*HSP60*), Kruppel-like factor 4 (*KLF4*), Cyclin-dependent kinase-9 (*Cdk9*), histone deacetylase 4 (*HDAC4*), SRY-Box transcription factor (*SOX6*), Frizzled class receptor (*FZD7*) and fibroblast growth factor receptor substrate 2 (*FRS2*) [124, 125]. In addition, it is connected with many transcription factors including; myocardin, Nkx2.5, serum response factor (SRF), Wnt pathway, fibroblast growth factor (FGF) pathway and Heart and Neural Crest Derivatives Expressed 2 (HAND2) [124, 125]. miR-1 influences the inflammatory cytokinase response through modulating KLF4 and NF-κB pathways as well as the TGF-β signalling pathway. miR-1 has been associated with a variety of cardiovascular conditions including: acute myocardial infarction [36–41], sudden cardiac death [42], microvascular obstruction leading to failed myocardial reperfusion [43], acute viral myocarditis [44], hypertrophic cardiomyopathy [45, 46], idiopathic dilated cardiomyopathy [47], hypertrophic obstructive cardiomyopathy patients undergoing trans-coronary ablation of septal hypertrophy (TASH) [48], the congenital heart malformation Tetralogy of Fallot [49], stress-related Takotsubo cardiomyopathy [50], hypertensive heart disease [51], geriatric patients with acute non-ST elevation myocardial infarction (NSTEMI) [52], acute coronary syndrome [53] and post-operative atrial fibrillation of coronary artery bypass patients [54, 55]. In contrast to upregulation of miR-1 in all of these cardiovascular conditions, consistent downregulation of miR-1 has been noted in heart failure [56–58], which indicates a diversity of roles of this microRNA in the process of cardiac injury.

### Hsa-miR-17

miR-17 forms part of a cluster of miRNAs’ (including miR-17-5p and -3p, miR-18a, miR-19a and b, miR-20a and miR-92a) with varied and significant roles in cancer and aging [126]. miR-17 has been shown to inhibit the transforming growth factor β (TGF-β) pathway which results in instability of atherosclerotic plaques in acute coronary syndrome [59] and coronary artery disease [60]. Another target of miR-17 is the connective tissue growth factor (CTGF) and thrombospondin-1 which affects myocardial fibrosis and significant expression of miR-17 has been linked to heart failure [61] and hypertrophic cardiomyopathy [62]. Increasing the activity of matrix metalloproteinases (MMPs) by miR-17 leads to breakdown of the extracellular matrix (ECM) which is a key factor in bicuspid aortic valve disorder [63].

### Hsa-miR-19a

miR-19a is strongly associated with several cancer types and even functions as an oncomir within the AKT-mTOR signalling pathway via silencing of the *PTEN* tumor suppressor gene [126]. Upregulation of miR-19a has been associated with reducing the levels of the bone morphogenetic protein receptor type II (BMPR2) linked to pulmonary arterial hypertension [64]. The *HMG-Box Transcription Factor 1* (*HBP-1*) gene is a known target for miR-19a which leads to an increase in macrophage migration inhibiting factor (MIF) that links miR-19a overexpression to both acute coronary syndrome [65] and atherosclerosis [66].

### Hsa-miR-29a

miR-29a is also part of a family of microRNAs’ which target a group of functionally related genes involved in apoptosis (*Tcd1*, *Mcl1*, *p85a*, *CDC42*, *YY1*, *CDK6*), cell differentiation (*YY1*, *HDAC4*), regulation of the extracellular matrix proteins (*Collegen* (*I*, *III*, *IV*), *LAMC1*, *FBN1*, *ELN*, *MMP2*, *ITGB1*) and immune responses (*B7-H3*, *Interferon-γ*) [127]. In cardiovascular diseases it has been linked to bicuspid aortic valve disorders [67], hypertrophic cardiomyopathy [68, 69, 62], coronary heart disease [70, 71], valvular heart disease [72], cardiac fibrosis [73], left ventricular remodelling [74] and pulmonary arterial hypertension [75]. The levels of circulating miR-29a are also thought to be linked to haemolysis of blood cells linked to certain cardiac pathologies rather than directly secreted only from the cardiomyocyte cells [128].

### Hsa-miR-34a

The miR-34 family has a variety of functions relating to cancer, particularly in the p53 tumor suppressor pathway [129] and it has been implicated in the processes of cardiac apoptosis, telomere attrition, DNA damage and inflammatory responses [130]. miR-34a influences lipid metabolism by inhibiting the Sirtuin 1 (SIRT1) pathway as well as stimulating pro-inflammatory cytokines such as IL-1b, IL-7A CRP and TNF-α which are strongly associated with cardiovascular diseases [76]. Links between miR-34a and several cardiovascular disorders have been well established including: chronic heart disease [76], cardiac aging [77], left ventricular (LV) remodelling [78], LV dysfunction [79], heart failure [80], acute myocardial infarction [81] and arterial fibrillation [82].

### Hsa-mir-122-5p

miR-122-5p is highly expressed within the liver where it is involved in lipid metabolism and hepatocyte homeostasis [131]. This may be a factor in the associations with lipid-related conditions such as coronary artery disease [83] and acute coronary syndrome [84]. Damage to hepatocytes was concluded to be the source of miR-122 from hypoperfusion resulting in significant expression during cardiogenic shock [85, 86], ventricular fibrillation sudden cardiac arrest [87] and chronic systolic heart failure [88]. miR-122 has been implicated in aortic valve dysfunctions through its mediating of tissue fibrosis and the extracellular matrix via the *TGFβR1* gene [89, 90]. It has also been associated with arrythmogenic cardiomyopathy [91], acute myocardial infarction [ [92]] and congestive heart failure [93].

### Hsa-miR-130a

miR-130a has been associated with apoptosis and angiogenesis. It has been found to be significantly expressed in the conditions of acute coronary syndrome [59] by acting on TNF-α, Toll-like receptors (TLR) and transcription factor NF-κb. The downregulation of the ERBB4 Tyrosine kinase receptor by miR-130 leading to increased left ventricle dilation and hypertrophy was found in the condition of peripartum cardiomyopathy [94]. It has also been linked to pulmonary hypertension [95], coronary heart disease [96] and aortic valve dysfunction [89].

### Hsa-mir-199a

mir-199a is widely expressed in the myocardium and is highly sensitive to oxygen tension and hypoxia [132]. It has been linked to Sirtuin 1 (SIRT1) which is a cardioprotective protein involved in the regulation of angiogenesis, endothelial function and vascular homeostasis. Downregulation of miR-199a has been noted in acute heart failure [97] and leads to an increase in SIRT1 expression in postoperative atrial fibrillation [98] and coronary heart disease [99–101]. Downregulated miR-199 also increased levels of atherosclerosis-related biomarkers (Angiogenin, Galactin-3 and Neuropilin-1) in heart failure patients with peripheral artery disease [102]. Increased miR-199 has also been associated to atrial fibrillation in HFrEF patients [103] and acute myocardial infarction [104].

### Hsa-miR-378a-3p

miR-378a has varied functions in metabolism, muscle development, inflammation and angiogenesis [133]. It is highly expressed by cardiomyocytes but evidence for a specific role in response to cardiac damage is still unclear [133]. Significant down-regulation of miR-378 has been noted in both coronary heart disease [105] and coronary artery disease [106].

### Hsa-mir-423

miR-423 has a functional role in cardiomyocyte apoptosis and has been linked to regulation of transcription factors of the *OGT* and *PA2G4* genes in evidence from animal and in silico models [107]. It is correlated with levels of the cardiomyocyte-secreted hormone NT-proBNP which is used widely as a diagnostic of heart failure [108, 109]. Dysregulation of miR-423 has been found to be highly variable between cardiac disorders and potentially subject to rapid changes. Decreased levels have been linked to poor clinical outcomes in acute heart failure patients [58, 110] and lower risk in coronary artery disease [111]. Whereas, significantly increased miR-423 has been found in many cardiac disorders including acute myocardial infarction [38, 107, 112], cardiogenic shock [113], dilated cardiomyopathy [108], left ventricular remodelling [114, 115], transposition of the great arteries [116] and heart failure [80, 109, 117].

### Hsa-mir-499

miR-499 is highly expressed in heart muscle and is released directly from the heart myocardium following tissue damage [40]. It has 70 primary mRNA targets involved in the developmental and metabolic pathways including SRY box 6 (Sox6), thyroid hormone receptor associated protein 1 (THRAP1), myocyte enhancer factor 2C (MEF2C), insulin-like growth factor-1 (IGF-1), pyruvate dehydrogenase subunit X (PDHX) and mediator complex subunit 13 (MED13) [134]. miR-499 also regulates the kinase/phosphatase pathways, βMHC (myosin heavy chain) isoform switching, phosphorylation of the signalling proteins HSP90β and PP1α, mitogen-activated protein kinase (MAPK) cascades, mRNA transcription via *Hipk1* and *Hipk2* regulation, Ca^2+^ transport and cell survival [134]. Increased expression of miR-499 has been found in acute myocardial infarction [38, 41, 118, 119], stable coronary artery disease [120], acute coronary syndrome [40, 121], ST-segment-elevation myocardial infarction (STEMI) [115], non-ST elevation myocardial infarction (NSTEMI) [52], dilated cardiomyopathy [122], heart failure with preserved ejection fraction (HFpEF) [123], sudden cardiac death [42] and unstable coronary artery disease [83].

### Hsa-mir-885-5p

Recently identified as a regulator of cardiomyocyte apoptosis in human cardiomyocytes through inhibition of the genes *PTEN*, *BCL2L11* and modulation of the AKT/mTOR signalling pathway [135]. miR-885 is also implicated in metastasis of certain cancers and as an indicator of toxic liver damage [136, 137] but no relevant clinical studies supporting the association of this microRNA with cardiovascular diseases were found.

### Hsa-miR-1273 g-3p

miR-1273 g has been linked to breast cancer [138, 139] and radiation treatments of cancer [140]. However, no clinical studies have reported a link with cardiovascular diseases and therefore, this microRNA is not currently considered informative for cardiotoxicity in breast cancer patients.

### Hsa-miR-4638-3p

No relevant clinical studies were identified to confirm the association of this microRNA with cardiovascular diseases.

## Discussion

Based on a systematic review of existing literature, 29 miRNA markers were identified for the investigation of chemotherapy induced cardiotoxicity (CIC) in breast cancer patients [28–35] (Table 1). However, a lack of replication of results amongst miRNA studies is highlighted as a major limitation of identifying informative miRNA markers, with many studies producing conflicting results or not supporting previously observed significant changes in miRNA expression. Therefore, for this review a strategy of grouping miRNAs by evidence of the type of replication was adopted whereby miRNAs were considered informative if they showed significant changes in expression in one or more studies and if they had been independently replicated but found to be non-significant in another study (Table 1, Sections A & B). miRNAs that have been found to be non-significant in one or more independently replicated studies were rejected (Table 1, Section C), as were miRNAs which were found to have conflicting directions of expression change in independently replicated studies (Table 1, Section D). The 14 shortlisted miRNAs were further examined in the literature for clinical studies of patients with cardiovascular diseases (Table 3) to identify corresponding cardiac conditions where these miRNAs have been found to have a significant change in expression. The direction of expression changes seen in these microRNAs were also replicated in a number of other cardiovascular diseases which confirms their utility as biomarkers of cardiac damage similar to that expected from cardiotoxicity (cardiomyocyte apoptosis, hypotrophy and fibrosis). However, three miRNAs were rejected for a lack of supporting evidence as no relevant publications in any cardiovascular conditions have reported findings for these markers. Therefore, the remaining 11 miRNAs (miR-1, 17, 19a, 29a, 34a, 122, 130a, 199a, 378a, 423 and 499) were concluded to be most suitable for the detection of cardiac damage resulting from exposure to chemotherapy agents.

Many of the miRNAs reported in the reviewed cardiotoxicity studies showed no significant changes between symptomatic and control groups which could indicate that either these markers were not activated within the pathway of cardiotoxic damage or that they may have been expressed at an earlier or later time point. The expression of miRNAs is known to change rapidly in some cardiomyopathies [74] and few of the studies included multiple sampling points. The majority of the clinical studies cited measured miRNAs immediately post-treatment or within 6 months of the end of treatment and only two studies took samples during the chemotherapy treatment period [28, 31]. The temporal variation in circulating microRNA expression may not necessarily be related to cardiotoxicity but to other comorbidities, functional pathways or patient-related factors [141]. Expression changes of miRNAs related to skeletal muscle and the cardiovascular system have been noted to be influenced by exercise for up to 24 hrs [142]. Dietary factors such as alcohol [143] and saturated fatty acids [144] can also influence miRNAs, in addition to the potential for homologous xenomiRs of plant [145] and animal [146] origin that can persist through the human digestive system. miRNA data is inherently noisy due to these exogenous factors which when combined with variability and uncertainties introduced by the methods of sample processing and analysis [147], make the use of such data very difficult and open to errors.

A major limitation of the studies listed in Table 1 is the small number of patients in the groups showing cardiotoxicity, between 7 and 20 subjects (Table 2), who were primarily classified by reduced LVEF (Left Ventricular Ejection Fraction) or elevated Troponin levels. This is reflected in the lack of repeatability between studies for the same microRNAs resulting in no significant changes in expression detected and a reduced statistical power for detecting dysregulation [148]. Small sample size reduces both the probability of detecting an effect and also that a statistically significant result reflects a true effect [149]. This is driven by the standard error of the measurements for miRNAs, the source of which may be interaction of a specific miRNA with different mRNA and gene pathways. Thus, it is essential to keep in mind that some miRNAs may suffer from a higher standard error, that cannot be adequately quantified or addressed with the small sample sizes found in most of the reviewed studies. Therefore, in addition to collating literature reporting statistically significant miRNA biomarkers associated to cancer treatment induced cardiotoxicity, we also looked at the change in level of expression of the reported miRNA in each group (cardiotoxic vs non-cardiotoxic). We expect true positive effects to replicate across independent studies, not just in terms of being statistically significant, but also in terms of the change of direction in the level of expression (increase versus decrease). Many of the attempted replications although reported as statistically significant in more than one scientific publication, in fact report opposite directionality in the level of expression in otherwise matching groups. This indicates that the quantification of this miRNA has a higher standard error and, therefore, is more prone to wide variations in measurements in groups of small sizes, resulting in false positive findings. An outcome of this manuscript is that there is a clear need to assess the standard error of each miRNA in terms of the replicability of measurements within a homogeneous group of patients. This value, along with all other required parameters (expected effect sizes, analytical approach, etc) should then be used to calculate the minimum sample sizes required for any study that considers that specific miRNA as a potential biomarker. Large sample sizes can offset high standard error in miRNAs, although to determine exactly how large these sample sizes need to be requires some assumptions in terms of the expected effect sizes and standard error in miRNA quantification. To address such issues, the co-authors of this manuscript are working through the CardioCare project funded under Horizon2020 to establish a large patient cohort (750 breast cancer patients determined based on statistical power analyses), that explores miRNAs as well as other potential biomarkers of cancer treatment induced cardiotoxicity with results expected in 2024 [150].

**Fig. 4:**
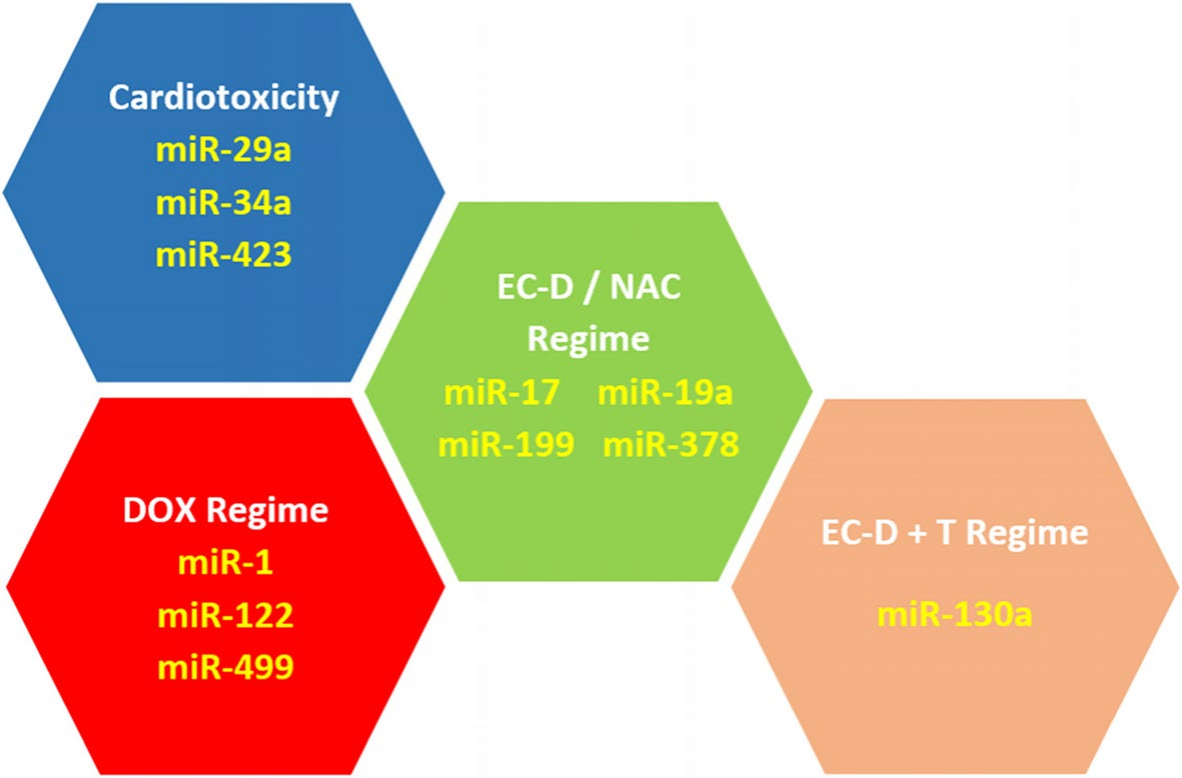
Panels of most-informative microRNA’s for chemotherapy-induced cardiotoxicity in breast cancer patients separated by treatment type. DOX = Doxorubicin, EC-D = Epirubicin + Cyclophosphamide & Docetaxel, NAC = Cyclophosphamide + Epirubicin & Paclitaxel, EC-D + T = Epirubicin + Cyclophosphamide & Docetaxel + Trastuzumab

Interpretation of the expression of one specific miRNA is difficult due to the potential confounding variables in patients with different characteristics, comorbidities and treatment regimes. Therefore, it is considered essential to apply a panel of several miRNAs linked to a condition of interest so that a profile of expression changes is generated, rather than relying on a single miRNA [151, 152]. Such panels have been developed previously for cardiovascular conditions such as myocardial infarction [153], dilated cardiomyopathy [151] and have been applied for prognostic and diagnostic purposes in other conditions including breast cancer [154], prostate cancer [155] and non-small cell lung cancer [156]. The nature of the chemotherapy agent appears to play a significant role in the expression of miRNAs as different agents elicited responses in different markers. The majority of studies have focused on anthracycline use and cardiotoxicity as this group of drugs are known to cause higher rates of cardiac damage than other chemotherapy agents [5]. Doxorubicin indicated significant changes in miRNA expression whereas, Epirubicin used as a single therapy did not [32]. Epirubicin is commonly used in conjunction with Cyclosphosphamide (alkylating agent) and Docetaxel or Paclitaxel (antimicrotubule agents) (termed EC-D or NAC regimes) and these treatments did result in dysregulation of several miRNAs with some differences to Doxorubicin. Only one study reported a significantly differentially expressed miRNA in patients undergoing an EC-D plus Trastuzumab monoclonal antibody therapy [35]. From the results presented here a panel of informative miRNA markers specific to each chemotherapy approach is suggested as shown in Fig. 4. Three miRNA markers; miR-29a, miR-34a and miR-423, are considered as general cardiotoxicity indicators and these should be supplemented by miR-1, miR-499 and miR-122 for patients undergoing a Doxorubicin treatment regime or miR-17, miR-19a, miR-199 and miR-378, for patients undergoing a EC-D regime. Additionally, for patients undergoing a Trastuzumab regime, miR-130a can be utilised. Thus, three panels of seven to eight miRNAs are suggested as the most effective approach to identify chemotherapy induced cardiotoxicity in breast cancer patients. Further research is required to investigate the prognostic value of each panel and the precise miRNA responses to each specific chemotherapy regime and prove clinical relevance of these panels.

miRNA modulation holds good promise as a therapeutic strategy to counteract cardiotoxicity induced by anticancer treatments. miRNAs are useful both as biomarkers of cardiotoxicity and for targeted therapy, since they may modulate entire signalling pathways. Unfortunately, many miRNAs modulated by anticancer treatments are also involved in cardiotoxicity. Therefore, the comprehension of the mechanisms elicited by miRNAs and the amelioration of specific delivery in either cardiac or tumor regions, could help to reduce negative side effects.

## Conclusion

The current body of evidence reveals that miRNAs can potentially offer clinically relevant information with regards to chemotherapy induced cardiotoxicity. However, many miRNAs reported as associated with these conditions may be the outcome of underpowered studies due to small sample sizes. This has led to poor replication of results between studies and limits the evidence for the application of miRNAs as clinical biomarkers. Through this work, we present a systematic review of relevant miRNA studies and a list of the most informative miRNAs based on independent replication, direction of significant expression change and additional evidence from clinical studies of each miRNA within the wider field of cardiovascular disease. The list of potential miRNA biomarkers to assess cardiotoxicity in cancer care are presented as a panel which can be modified to the therapeutic approach under consideration. We recommend more studies with sufficient statistical power to accurately evaluate the potential use of miRNAs in clinical care. Statistical power needs to be assessed based on empirically quantified parameters for each miRNA considered.

## Data Availability

Not applicable.
